# CD6 in Human Disease

**DOI:** 10.3390/cells14040272

**Published:** 2025-02-13

**Authors:** Mikel Gurrea-Rubio, David A. Fox, Javier S. Castresana

**Affiliations:** 1 Division of Rheumatology, Department of Internal Medicine, University of Michigan, Ann Arbor, MI 48109, USA; dfox@umich.edu; 2 Department of Biochemistry and Genetics, University of Navarra School of Sciences, 31008 Pamplona, Spain

**Keywords:** CD6, CD318, CD166, CD44, immunotherapy, autoimmunity

## Abstract

CD6 is a cell surface protein expressed by T cells, a subset of NK cells, a small population of B cells, and thymocytes. CD6 has multiple and complex functions due to its distinct functional epitopes that mediate interactions with several ligands including CD166 (ALCAM) and CD318 (CDCP1). An additional molecule, CD44, is being investigated as a potential new ligand of CD6. CD6 plays critical roles in lymphocyte activation, proliferation, and adhesion to antigen-presenting, epithelial, and cancer cells. CD6 is a risk gene for multiple autoimmune diseases, possibly related to its numerous roles in regulating CD4+T-cell responses. Additionally, CD6 is a potential target for cancer immunotherapy. Here, we dissect the role of CD6 in the pathogenesis of more than 15 diseases and discuss recent data supporting the use of CD6-targeted therapy in humans.

## 1. CD6

CD6 is a type I transmembrane glycoprotein expressed by T-lymphocytes, a subset of NK cells (CD56dim), and a subset of B-lymphocytes (B1a). CD6 expression on immature thymocytes is generally low but increases as the thymocytes mature, with the highest expression on mature single-positive thymocytes (CD4 or CD8) [1,2,3,4,5,6]. Although it is believed that no cells outside of the immune system express CD6, some reports have shown evidence of CD6 expression at the mRNA level in various regions of the brain, specifically in the cortex, basal ganglia, and cerebellum. Difficulty in confirming protein expression and low mRNA expression of CD6 in brain tissue is consistent with infiltrating T cells and B cells in the brain as the source of CD6 expression [3].

CD6 consists of a transmembrane region, a cytoplasmic tail for intracellular signaling, and an extracellular portion with three scavenger receptor cysteine-rich (SRCR) domains. The three SRCR domains (highly conserved across species) are responsible for its interactions with several CD6 ligands. CD318 is also referred to as CUB domain-containing protein 1 (CDCP1) [7] and CD166 is known as the activated leukocyte cell adhesion molecule (ALCAM) [8]. Furthermore, recent research has found strong biochemical and biophysical evidence supporting CD44 as a new ligand for CD6 [9]. All three CD6 ligands are ubiquitously expressed on many cell types, including endothelial, epithelial, and cancer cells, including cancer stem cells [10,11]. Moreover, β-galactoside-binding lectins Galectin 1 and 3 (Gal-1 and Gal-3) are also known to interact with CD6 and influence various aspects of T-cell function such as adhesion, activation, and apoptosis [12].

## 2. Functions of CD6

CD6 plays important but incompletely understood roles in T-cell activation, signaling, cell adhesion, the regulation of immune responses, and the formation of the immune synapse. Early evidence for the involvement of CD6 in T-cell activation came from work with monoclonal antibodies (mAbs) aimed at CD6, which triggered T-cell activation and proliferation [13]. Many studies have further substantiated CD6’s co-stimulatory role, through enhancement of T cell receptor (TCR) signaling [14,15,16]. However, studies suggest that CD6 also acts as an attenuator of early and late T cell responses [17,18]. The role of CD6 in T-cell signaling may depend on the balance between stimulatory and inhibitory signals that are transmitted when CD6 is engaged by its various ligands. CD6 is also involved in cell adhesion by facilitating the attachment of T-lymphocytes to thymic epithelial cells [19], blood–brain barrier cells [20], antigen-presenting cells [21], keratinocytes, and synovial fibroblasts [22,23]. These interactions are likely to be important in both normal and autoimmune T cell responses.

CD6 regulates B1a cell function and contributes to the production of natural IgM antibodies, which are critical for early immune responses against bacterial infections [24]. Recent work by Català et al. demonstrated that CD6^−/−^ mice have lower survival rates and higher bacterial levels after sepsis. These mice also exhibited reduced levels of natural polyreactive antibodies along with a decrease in cell counts of spleen B1a and marginal zone B cells. Their studies also showed that the adoptive transfer of wild-type cells and serum to CD6^−/−^ mice improves their survival after sepsis, highlighting CD6’s important role in early responses to bacterial infections [25].

Similar to CD6, cluster of differentiation 5 (CD5) is involved in the development, differentiation, activation, and survival of lymphocytes. CD5 and CD6 have structural similarities that include an extracellular region with three SRCRs, a transmembrane region, and cytoplasmic sites for phosphorylation and association with downstream signaling effectors. CD5 and CD6 physically associate with the T cell receptor (TCR) complex with which they co-localize at the center of the immunological synapse [26], providing inhibitory (CD5) and activating/inhibitory (CD6) signals [27].

## 3. Ligands of CD6

CD6 interactions with its ligands—CD166/ALCAM, CD318, and CD44—are important in the pathogenesis of various autoimmune diseases and cancer. CD166 is expressed by a great variety of cell types, including activated T cells, monocytes, epithelial cells, fibroblasts, and cancer cells. CD166 has been implicated in the pathogenesis of lupus nephritis [28], rheumatoid arthritis [29], Sjogren’s syndrome [30], and inflammatory bowel disease [31]. CD166 expression is tightly associated with worse prognosis and increased metastatic potential in several forms of cancer, including liver [32], thyroid [33], head and neck [34], and breast cancer [35,36,37]. Elevated CD166 expression is not, however, a uniform biomarker for a poor prognosis in cancer. For instance, in Ewing sarcoma, CD166 over-expression correlates with better outcomes and lower metastatic potential [38]. CD166 expression is also associated with improved prognosis and extended survival in patients with non-small-cell lung cancer [39]. CD6 transcript levels in colon cancer are inversely associated with disease progression [40].

CD318, expressed by the epithelium of normal and cancer cells, is involved in inflammatory responses, autoimmunity, and cancer. CD318 over-expression is tightly linked to unfavorable outcomes in many cancers, including breast [41], lung [42], colorectal [43], ovarian [44], renal [45], prostate [46], and pancreatic [47] cancers. Thus, in contrast to CD166, over-expression of CD318 is infrequently linked to a good prognosis in cancer. Nonetheless, this might not hold true for esophageal cancer. Research conducted by Sawada et al. revealed that the reduction in CD318 expression increases invasiveness and results in poor prognosis in esophageal cancer [48]. Importantly, CD318^−/−^ mice show resistance to the onset of several mouse models of autoimmunity [7,49]. In addition, recent research has shown that targeting the CD6/CD318 axis with the mAb UMCD6 (anti-CD6) increases cytotoxic-lymphocyte-mediated cancer cell death of breast, prostate, and lung cancers both in vitro and in vivo [39,50]. CD44, a new ligand for CD6 [9], is also expressed on cancer cells as well as many non-malignant cell types. CD44 plays a major role in regulating cancer cell proliferation and metastasis [51]. Precise information is not yet available concerning which CD44 isoforms and associated signaling pathways are engaged by CD6.

In addition, CD318 and CD44 are compelling markers for cancer stem cells of many solid malignancies such as breast cancer and prostate cancer. In light of these findings, current research is investigating how the interruption of CD6 interactions with its ligands can be utilized as a novel immunotherapy for cancer [52,53].

**Figure 1:**
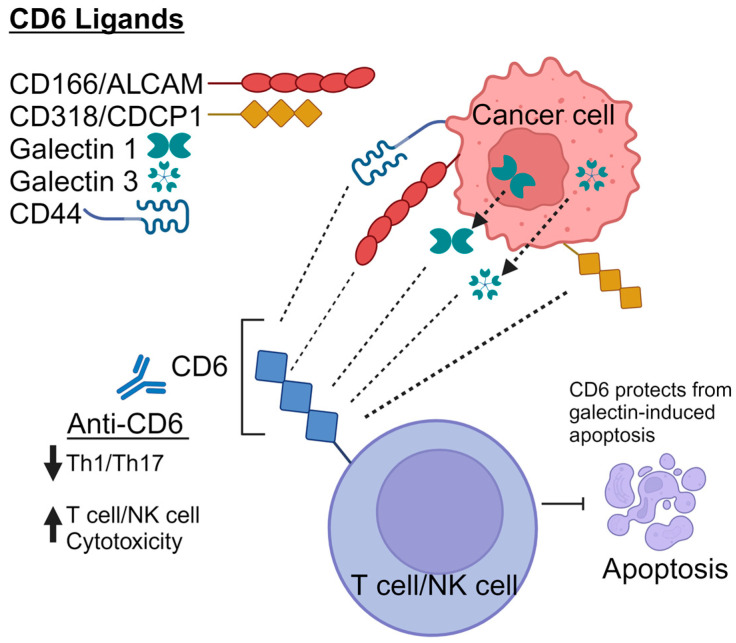
Schematic representation of endogenous CD6 ligands. CD6 is composed of three extracellular scavenger receptor cysteine-rich domains allowing for interactions with CD166 (ALCAM), CD318 (CDCP1), CD44, and Galectins 1 and 3. The interactions between CD6 and its ligands CD166 and CD318 can be targeted using monoclonal anti-CD6 antibodies, with the potential to be used in both cancer immunotherapy and to treat autoimmune diseases. T cells that express CD6 are protected from apoptosis induced by Galectins 1 and 3.

Galectins constitute a protein class that bind to β-galactose-containing glycoconjugates, fulfilling essential functions in developmental, homeostatic, and pathological processes. Thus far, there are 15 known galectins in mammals, all of which share a conserved protein fold within their respective carbohydrate recognition domains (CRDs) [54]. Galectins are categorized into three classes based on structure: prototype, chimeric, and tandem-repeat types. Escoda-Ferran et al. were pioneers in demonstrating that both Galectin 1 (Gal-1) and Galectin 3 (Gal-3) interact with CD6 and CD166/ALCAM, interfering with superantigen-induced T-cell proliferation, adhesion, and migration [12]. Furthermore, they found that CD6 expression protects T cells from Galectin-1- and 3-induced apoptosis. Both Gal-1 and Gal-3 are broadly expressed by fibroblasts, cancer cells, T and B cells, dendritic cells, monocytes, and neutrophils. Galectin 1 is important in cancer as it suppresses immune functions in tumor environments, aiding tumors in avoiding immune detection [55]. Galectin 3 is often elevated in cancer and is linked to tumor growth and spread, indicating a worse prognosis for patients with high levels [56]. The exact mechanism by which CD6 protects T cells from galectin-induced death is unclear. However, the relationship between galectins and CD6 could be significant for developing new treatments for diseases, especially cancer (Figure 1).

## 4. CD6 in Human Disease

### 4.1. Multiple Sclerosis

Multiple sclerosis (MS) is a long-term, immune-mediated disease of the central nervous system, usually characterized by widespread areas of demyelination in the brain and spinal cord. In MS, autoreactive T cells target myelin antigens, leading to neurodegeneration. The CD6 gene, like many other genes, can be alternatively spliced, resulting in multiple CD6 isoforms that affect how CD6 is targeted at the immunological synapse. T-cell activation is known to be influenced by the alternative splicing of exon 5, which is controlled by SRSF1, SRSF3, and hnRNPA1. In connection with this, recent research has identified CD6 as a gene associated with the risk of this disease. In a meta-analysis of genome-wide association studies regarding MS, Kofler et al. discovered a single nucleotide polymorphism, rs17824933, in the CD6 gene that serves as a genetic risk locus for MS. This polymorphism correlates with reduced expression of full-length CD6 in CD4+ and CD8+ T cells, resulting in diminished proliferation during long-term activation. Their studies also found that exon 5—which encodes for the extracellular binding site of the CD6 ligand CD166, and is required for CD6 stimulation—is consistently under-expressed by CD4+ T cells from individuals that carry the risk allele [57].

Several years after the discovery of CD6 as a risk locus for MS, Li et al. demonstrated that CD6-deficient mice (CD6^−/−^) display decreased pathogenic Th1 and Th17 cells, reduced T-cell infiltration in the spinal cord, and diminished disease severity in experimental autoimmune encephalomyelitis (EAE), a mouse model of MS [58]. Moreover, CD6-negative cells isolated from these mice exhibited augmented activation but reduced survival and proliferation, leading to decreased Th1 and Th17 polarization. In addition, a mouse anti-human CD6 monoclonal antibody (UMCD6) successfully treated EAE without depleting T cells in CD6-humanized mice [58]. These results suggest that CD6 is a negative regulator of T-cell activation, a positive regulator of activated T-cell survival/proliferation, and a potential new target for treating MS. An IgM anti-CD6 antibody has already been tested in MS but the small size of the uncontrolled study made it impossible to determine its therapeutic efficacy [59].

Recent evidence has also pointed out that the development of MS may be influenced by the CD6 ligands. Cayrol et al. demonstrated that CD166 expression on blood–brain barrier (BBB) cells is upregulated in both active MS and experimental autoimmune encephalomyelitis lesions. Moreover, blocking CD6 expression restricts the transmigration of CD4+ lymphocytes and monocytes across BBB endothelium and reduces the severity of EAE, suggesting that targeting ALCAM could be a potential therapeutic strategy for MS by limiting immune cell infiltration into the central nervous system [20].

### 4.2. Behçet’s Disease

Behçet’s disease (BD) is an autoimmune vasculitis disease of unknown cause. It is marked by the presence of oral and genital ulcers, uveitis, and skin lesions. Recent research suggests that certain variations in the CD6 gene are associated with increased susceptibility in the Chinese Han population. In a two-stage association study of Single-Nucleotide Polymorphisms (SNPs) of adhesion molecules in BD patients, Zhang et al. observed a markedly increased occurrence of the CT genotype, along with a decreased occurrence of the CC genotype and C allele of CD6 rs11230563 in BD patients compared to healthy controls [60]. These genomic studies of polymorphisms in BD suggest that variations in the CD6 gene may contribute to the development of BD and influence immune cell activation and infiltration into affected tissue, although functional genomic studies are needed to test such hypotheses.

### 4.3. Psoriasis

Psoriasis is a chronic inflammatory disease characterized by excessive epidermal growth that leads to the formation of red, scaly patches on the skin, usually on the elbows, knees, scalp, and trunk. Psoriasis is believed to be driven by a strong genetic component. Historically, Th1 cells were considered the main contributor to psoriasis but recent research has shown that Th17 cells—which produce interleukin-17 (IL-17)—are highly prevalent in psoriatic lesions and ultimately play a crucial role in driving the inflammatory cascade by stimulating keratinocyte proliferation and cytokine production [61]. Not surprisingly, CD6 has been found to play a crucial role in the progression of this disease. Consuegra-Fernandez et al. were the first group to demonstrate the role of CD6 in the pathogenesis of psoriasis. Their experiments showed that CD6-deficient mice have lower psoriasis-like skin inflammation, decreased epidermal thickness, and localized decreased production of pro-inflammatory cytokines, specifically IL-17. Furthermore, they conducted a retrospective analysis of 304 patients with psoriasis and found several CD6-associated genetic variations (rs17824933, rs11230563, and rs12360861) linked to more severe forms of psoriasis [62].

Itolizumab, an anti-CD6 humanized IgG1 monoclonal antibody that binds to domain-1 of CD6, is currently being used to treat psoriasis in India. Itolizumab’s mechanism of action selectively inhibits T-cell proliferation and cytokine production, and it especially affects IL-17 in producing CD4+ T cells (Th17), known to be involved in many autoimmune disorders like psoriasis [63]. The very first phase II studies involving itolizumab found significant improvement in psoriasis severity index scores in patients receiving itolizumab once every 2 weeks [64]. A phase III study further confirmed the efficacy of this antibody in treating moderate-to-severe psoriasis, with rapid improvement in psoriasis severity scores and no significant adverse events [65]. Mechanistically, phase II and III studies demonstrated that itolizumab can successfully reduce T-cell proliferation and lower the amount of IFN-γ secreted by T cells. Upon examination of the cytokine profile, these studies showed that pro-inflammatory cytokines are reduced following itolizumab treatment.

### 4.4. Atopic Dermatitis

Often referred to as eczema, atopic dermatitis (AD) is a chronic disease that causes inflammation, irritation, and redness of the skin. The exact causes that drive AD are not well-understood; however, like psoriasis, AD seems to have a strong genetic component. Several studies suggest that an imbalance in Th1/Th2 cells and skin barrier dysfunction contribute to AD pathogenesis, with Th17 and Th22 inflammatory responses playing a significant role [66]. A recent study using proteomics to identify blood markers for atopic AD found that CD6 protein levels in serum are significantly elevated in AD patients compared to psoriasis patients and healthy controls, thus suggesting that CD6 could be a potential biomarker specific to AD [67,68]. This finding aligns with earlier research showing increased levels of CD5 in AD, and supports the idea that dysregulated immune cell signaling—particularly involving CD6 and CD5—might play a role in the systemic nature of AD [69].

### 4.5. Asthma

Asthma is a chronic respiratory condition characterized by inflammation and reversible narrowing of the airways (bronchi). In this disease, patients present with excessive infiltration of inflammatory cells such as eosinophils, macrophages, and T cells in the bronchi [70]. Not surprisingly, Koziol-White et al. found that CD6 is highly expressed in patients suffering from fatal asthma. Next, their studies found that CD6+ lymphocytes can modulate the tension in the walls of the bronchi (bronchomotor tone) through actin cytoskeletal rearrangements in human airway smooth muscle (HASM), providing strong evidence that the inflammatory environment may influence CD6-dependent changes in bronchomotor tone [71]. Currently, a phase Ib randomized, double-blind, placebo-controlled study (known as EQUIP) is examining the safety and tolerability of itolizumab in patients with uncontrolled asthma as well as its pharmacokinetics, pharmacodynamics, and clinical activity.

### 4.6. Rheumatoid Arthritis

Rheumatoid arthritis (RA) is another chronic T-cell autoimmune disease that affects the joints, causing pain and swelling. RA can also affect many other organs such as the skin, heart, eyes, and brain. Although the exact cause of RA is unknown, the disease encompasses several mechanisms including T cells, which differentiate into Th1 and Th17 cells promoting inflammation; and B cells, which present antigens and produce autoantibodies against citrullinated proteins. Synovial lymphocytes, macrophages, and fibroblasts accumulate in the RA joints and secrete inflammatory cytokines such as IL-17, TNF-α, IL-6, and IFN-γ [72,73]. CD6 might play a crucial role in the progression of this disease by promoting the adhesion and activation of T cells within the inflamed joint, primarily through its interaction with its ligands CD166 and CD318. The interaction between CD6 and its ligands is vital for the initiation and progression of collagen-induced arthritis (CIA), which serves as a mouse model for RA [74]. CD6^−/−^ mice are protected from CIA, and collagen-specific Th9 and Th17 cells—as well as their corresponding inflammatory cytokines—are decreased in these mice. Similarly, CD6-humanized mice treated with UMCD6 (anti-CD6) displayed a similar reduction in joint inflammation and lower Th1/Th17 cells in CIA.

In humans, CD6 ligands are found in RA synovial tissue and are involved in T-cell adhesion to fibroblast-like synoviocytes. In addition, soluble CD318, found in RA synovial fluid, is chemotactic for T cells [7]. Results from a phase I clinical trial of the anti-CD6 antibody itolizumab suggest a clinical benefit without lymphopenia in RA patients [75].

### 4.7. Uveitis

Similar to rheumatoid arthritis, autoimmune uveitis (EAU) is another chronic autoimmune disease in which CD6 is believed to be involved in its pathogenesis. Zhang et al. examined the role of CD6 in a mouse model of EAU and found that CD6^−/−^ mice had significantly decreased retinal inflammation and reduced autoreactive T cell responses compared to wild-type mice [49]. In addition, their experiments confirmed the impaired uveitogenic capacity of T cells from CD6^−/−^ mice and also demonstrated that UMCD6 can reverse EAU in CD6-humanized mice.

### 4.8. Giant Cell Arteritis

Giant cell arteritis (GCA) is a debilitating inflammatory disease affecting the large blood vessels of the scalp, eye, and aortic branches. The CD6 locus is hypomethylated in the vasculitic lesions compared to normal controls, suggesting increased expression and potential involvement in the inflammatory process of this disease [76]. While this indicates a potential role for CD6 in GCA, further studies are required to fully understand the exact mechanisms and potential therapeutic implications.

### 4.9. Sjogren’s Syndrome

Sjogren’s syndrome (SS) is an autoimmune disorder of the exocrine glands that primarily impacts the lacrimal and salivary glands, resulting in dryness of the mouth and eyes. More widespread systemic inflammatory complications are not infrequent. While historically considered primarily a T-cell-mediated disease, recent research indicates that both T cells and B cells are essential in the pathogenesis of this disease. Alonso et al. found that CD6 is absent on transitional B cells and present on mature and memory B cells. The proportion of CD6-positive cells in B cells is decreased in patients with primary SS compared to those with rheumatoid arthritis. This reduction is not due to CD6 shedding from B cell membranes but from the lowering of memory B lymphocytes, possibly due to migration into lesional tissues. Alonso et al. also found that CD166 is highly expressed on epithelial cells from the salivary glands of patients with SS [77]. Itolizumab has been proposed for clinical trials in SS [78].

### 4.10. Inflammatory Bowel Disease 

Inflammatory bowel disease (IBD) is an idiopathic disease that causes chronic inflammation of the digestive tract. The pathogenesis of IBD is not yet well-understood. However, continuous and recurring intestinal inflammation as a result of exacerbated immune responses—which are known to be influenced by genetic and environmental factors—are thought to lead to or increase susceptibility to IBD. To date, numerous studies have identified genetic variants related to this disease.

Polymorphisms in CD6 are associated with an increased risk of IBD. In a recent study, Casadó-Llombart et al. analyzed DNA samples from IBD patients and tested the association of three CD6 single nucleotide polymorphisms (rs17824933, rs11230563, and rs12360861) with susceptibility to and the clinical parameters of Crohn’s disease (CD). Their analyses showed a strong association of CD6 variation with CD ileal location (rs17824933^G^) and poor prognosis (rs12360861^G^) [31]. In connection with this, Ma et al. also observed that CD6 and CD166 expression is increased in the inflamed mucosa of IBD patients when compared to healthy controls; furthermore, their data showed a strong correlation between CD6 expression and disease activity in IBD patients [79].

### 4.11. Graft-Versus-Host Disease

Graft-versus-host disease (GVHD) is a complex immunological disorder that occurs when donor immune cells attack the recipient’s tissues after a bone marrow transplant, often leading to serious complications across multiple organ systems. Rasmussen et al. provided the first hint linking CD6 and alloreactivity; their studies identified a subset of peripheral blood T cells with low or no CD6 (CD3+CD5intCD6lo/−) in the blood of healthy participants and proved that CD6lo/− T cells were less reactive to allogeneic stimulation but not to soluble recall antigens (tetanus toxoid) or mitogenic lectins (PHA) [80]. In addition, Soiffer et al. provided the first hint that CD6 might be involved in the pathogenies of GVHD [81]; their studies showed that depleting CD6+ cells with an anti-CD6 IgM monoclonal antibody (anti-T12) prior to hematopoietic transplantation reduced the incidence of acute and chronic GVHD and obviated the need to administer immune suppressive medications. Moreover, in a humanized xenograft model of GVHD, Soiffer et al. further demonstrated that administration of itolizumab significantly reduced mortality and tissue-infiltrating CD3+T cells in major organs.

In another study conducted by Schuster et al., a novel haploidentical transplant method—that employs unmanipulated bone marrow followed by CD6-depleted peripheral blood stem cells—was found to decrease relapse and mortality in children suffering from refractory acute leukemia or experiencing relapse following stem cell transplantation [82]. More recently, Rambaldi et al. found that CD6 expression was reduced in regulatory T cells and CD8+T cells in acute GVHD compared to healthy donors [83]. Their work further demonstrated that itolizumab inhibited CD4 and CD8+T-cell activation and proliferation in pre-GVHD samples but inhibition was less prominent in samples collected after acute GvHD onset. Importantly, a newly developed CD6-targeted antibody–drug conjugate (CD6-ADC) using the monoclonal antibody UMCD6 has been found to efficiently treat two pre-clinical models of GVHD [84]; this study suggests that CD6-ADC could be developed as a potential therapeutic agent for selectively eliminating pathogenic T cells and treating GVHD.

### 4.12. COVID-19

Although most patients with COVID-19 present mild symptoms, some develop severe inflammation-related complications that can lead to death. One of the possible mechanisms that contributes to rapid disease progression is the appearance of inflammatory cytokine-release syndromes (also known as cytokine storms). Some evidence has been provided to suggest therapeutic activity of anti-CD6 in decreasing the hyperinflammatory status in COVID-19 patients. Data from five studies reported no fatalities when itolizumab was added to best supportive care (BSC) [85]. Patients who received itolizumab and BSC showed improvements in oxygen saturation and a robust decrease in two or more inflammatory cytokines (e.g., IL-6 and TNF-α) when compared with BSC alone [86]. Similarly, single-arm trials of itolizumab reported a decrease in IL-6 production and lower mortality in hospitalized COVID-19 patients with a satisfactory safety profile [87].

### 4.13. Lupus Nephritis

Newer concepts regarding the pathogenesis of lupus nephritis (LN), a frequent complication of systemic lupus erythematosus (SLE), incorporate a role for T cells. Multiple studies now indicate that CD6 and its ligand CD166 (ALCAM) play significant roles in lupus, particularly in its development. Chalmers et al. studied CD6/CD166 expression in the kidneys of lupus nephritis (LN) patients and tested whether soluble CD166 in urine could be used as a disease biomarker [28]. They found that CD6 is exclusively expressed in T cells, whereas CD166 is found in resident and infiltrating renal cells. Moreover, CD6+ T cells were found in higher proportions in patients with LN in comparison to healthy controls, particularly in patients with the most severe forms of kidney damage. Importantly, soluble CD166 in urine was significantly elevated in patients with active LN irrespective of ethnicity. Importantly, a CD6 blockade alleviated mouse models of LN by decreasing glomerulonephritis, inflammatory markers, and disease measures. Itolizumab has completed phase I trials for LN and SLE; phase II trials are currently underway.

### 4.14. Diabetes

The CD6/CD318 axis is currently being investigated for its potential involvement in type 1 diabetes (T1D), particularly in modulating the activation of autoreactive T cells that attack insulin-producing beta cells in the pancreas. CD318 expression was previously believed to be restricted to mesenchymal-epithelial cells, contributing to CD6-mediated T-cell activation within CD318-expressing tissue. However, Do et al. recently found that CD318 can be induced by activation in a subset of human monocytes exposed to lipopolysaccharide (LPS) and IFN-γ, as well as in a subpopulation of human CD318+ myeloid dendritic (mDC) cells [88]. Importantly, recombinant CD318 suppressed T-cell function and CD318+ DCs hindered the proliferation of autoreactive T cells specific for GAD65, a target in T1D. These findings, for the first time, allude to a possible unique immunoregulatory role for the CD6 ligand CD318 in T-cell-mediated autoimmune disorders, as well as a possible novel immunological checkpoint relevant to the pathogenesis and treatment of T1D.

The use of antibodies targeting CD6 as a potential treatment strategy for T1D is currently being explored by multiple scientific groups. In a study conducted by Schneider et al., short-term treatment with T1h (a well-known humanized IgG1 anti-CD6 monoclonal antibody) and oral insulin protected newly diabetic mice from developing diabetes; however, the mice became diabetic again shortly after stopping the treatment [89]. In addition, CD6-CAR Treg cells, a type of engineered regulatory T cells (Tregs), are currently being studied as a potential new treatment for T1D [90].

### 4.15. Lymphoid Malignancies

Lymphoid malignancies are a broad group of neoplasms with different clinical presentation, histology, and biology. T-cell and NK-cell lymphomas are rare lymphomas that usually display aggressive clinical progression. On the other hand, B-cell lymphomas are significantly more prevalent—accounting for approximately 80% of individuals with non-Hodgkin lymphomas—and tend to have better prognosis [91]. Regarding CD6, a recent retrospective study by Zhao et al. reported increased CD6 expression in aggressive NK-cell leukemia/lymphoma (ANKLL) and extranodal NK/T-cell lymphoma (ENKTL) patients [92]. In addition, Parameswaran et al. recently verified that human T-cell lymphoma cell lines express CD6 at high levels and found that a CD6-targeted antibody–drug combination (termed anti-CD6-ADC) reverses the growth of tumors in vivo and specifically kills T-cell lymphoma cells in vitro, suggesting that CD6 could be a good marker for the treatment of these very aggressive T-cell lymphomas [93].

### 4.16. Solid Tumors

While CD6 itself is primarily expressed on immune cells, its ligands are frequently expressed on tumor cells. This makes CD6 a potential target for cancer immunotherapy. Targeting CD6 enhances lymphocyte-mediated anti-tumor immunity. UMCD6—an mAb targeting the CD6/CD318 axis—enhances the ability of CD8+T, NK-T, and NK cells to kill breast, prostate, and lung cancer cells, accompanied by robust changes in gene and protein expression of the activating receptor NKG2D and the inhibitory receptor NKG2A [39,50,94]. Importantly, UMCD6 is known to block T-cell-dependent autoimmunity through effects on differentiation of effector CD4+ T cell subsets, opening a new approach to cancer immunotherapy that will suppress rather than instigate immune-adverse related events [95].

Chimeric antigen receptor (CAR) T-cell therapy is transforming the treatment of many types of hematological malignancies and solid tumors. Cancer-related ligand receptors, such as CD166, CD318, and CD44, are currently being explored for the treatment of many solid tumors. A recent study led by He et al. has shown that CD6-augmented CAR-T cells exhibited potent cytotoxicity targeting colorectal cancer (CRC) stem cells, making this targeted therapy a promising approach for CRC therapy [96]. Moreover, CAR-T cells targeting CD166 (CD166.BBζ CAR-T cells) have shown great promise in eliminating CD166+ osteosarcoma cells in vitro and in vivo [97].

## 5. Conclusions—The Potential of Targeting CD6 in Human Disease

We have reviewed the role of CD6 in the pathogenesis of autoimmune diseases and shown that aggravation of autoimmune inflammation is partly mediated through CD6. Although much remains unknown about the role of CD6 in T-cell activation, the new data from human autoimmune disease databases and experimental systems argue for testing anti-CD6 as a therapy for the treatment of a wide range of autoimmune syndromes, specifically those that are mediated by Th1 and Th17 cells. Itolizumab and CD6 CAR-T cells are currently being evaluated in multiple clinical trials in patients with severe diseases, including GVHD, lupus nephritis, and uncontrolled asthma (Table 1). Moreover, the strong expression of CD6 ligands on cancer cells suggests that lymphocyte-engaging therapies such as anti-CD6 should be advanced into clinical trials based on the findings that CD6 blockade concurrently reduces T-cell autoimmunity and increases T-cell and NK-cell cytotoxicity.

## Figures and Tables

**Table 1:** Current clinical trials targeting CD6.

Clinical Trial	Treatment	Phase	Condition
NCT05993611	CD6-CAR T regs	Ib	Chronic graft-versus-host disease after allogeneic hematopoietic cell transplantation
NCT04007198	Itolizumab (anti-CD6)	Ib	Moderate-to-severe uncontrolled asthma
NCT04128579	Itolizumab (anti-CD6)	Ib	Systemic lupus erythematosus with or without active proliferative nephritis
NCT04475588	Itolizumab (anti-CD6)	II	COVID-19 complications
NCT03763318	Itolizumab (anti-CD6)	I/IIb	Acute graft-versus-host disease in combination with corticosteroids
NCT05263999	Itolizumab (anti-CD6)	III	Acute graft-versus-host disease in combination with corticosteroids

## Data Availability

Not applicable.
